# CCR2 is localized in microglia and neurons, as well as infiltrating monocytes, in the lumbar spinal cord of ALS mice

**DOI:** 10.1186/s13041-020-00607-3

**Published:** 2020-04-29

**Authors:** Hiroyasu Komiya, Hideyuki Takeuchi, Yuki Ogawa, Yuki Hatooka, Keita Takahashi, Atsuko Katsumoto, Shun Kubota, Haruko Nakamura, Misako Kunii, Mikiko Tada, Hiroshi Doi, Fumiaki Tanaka

**Affiliations:** Department of Neurology and Stroke Medicine, Yokohama City University Graduate School of Medicine, 3-9 Fukuura Kanazawa-ku, Yokohama, 236-0004 Japan

**Keywords:** Amyotrophic lateral sclerosis, CCR2, Astrocyte, Neuron, Microglia, Monocyte

## Abstract

It remains controversial whether circulating monocytes expressing CCR2 infiltrate the central nervous system (CNS) and contribute to pathogenicity of amyotrophic lateral sclerosis (ALS). A previous report used conventional immunohistochemistry to show that CCR2 is exclusively expressed by astrocytes, but not infiltrating monocytes/microglia or neurons, in the spinal cords of ALS model mice. In this study, we assessed the cellular distribution of CCR2 in the CNS of ALS mice using CCR2-reporter mice (*Ccr2*^*rfp/+*^-*Cx3cr1*^*gfp/+*^-SOD1^G93A^ Tg mice), a more sophisticated method for directly detecting the distribution of CCR2 protein. We found that infiltration of CCR2^+^ monocytes in the lumbar spinal cord increased over the course of disease progression. Moreover, from the middle stage of disease, CCR2 was partially distributed in microglia and neurons, but not astrocytes, in striking contrast to the previous findings. These novel observations suggested that CCR2^+^ monocyte infiltration leads to CNS environmental deterioration due to toxic conversion of microglia and neurons, creating a vicious cycle of neuroinflammation and leading to acceleration of ALS pathology. Our findings also show that this reporter mouse is a useful and powerful tool for obtaining new insights into the pathomechanisms of ALS.

## Main text

Amyotrophic lateral sclerosis (ALS) is a fatal neurodegenerative disease characterized by the selective loss of motor neurons in the central nervous system (CNS). The non-autonomous neuronal death hypothesis, based on recent studies, states that neuroinflammation by nonneuronal neighboring cells, such as glia and infiltrating cells, is critical for disease progression in ALS [1]. In general, tissue inflammation induces migration of circulating monocytes, especially chemokine CC motif receptor 2 (CCR2)^high^ monocytes [2]. It remains controversial whether CCR2^high^ monocytes infiltrate the CNS and contribute to ALS pathogenesis [3, 4]. However, Kawaguchi-Niida et al. showed using conventional immunohistochemistry, that CCR2 is exclusively expressed by astrocytes, but not infiltrating monocytes/microglia or neurons, in the spinal cords of ALS mice [5]. This variability on CCR2^+^ cellular distribution may partly depend on how CCR2 is detected. For immunostaining, commercially available anti–mouse CCR2 antibodies are usually suitable for flow cytometry, but less so for immunohistochemistry/immunofluorescence, as is usually the case for cell surface antigens. Accordingly, the CCR2-RFP reporter mouse was developed to circumvent this difficulty [2].

In this study, we used this CCR2-reporter mouse to directly detect the distribution of CCR2 protein, with the goal of assessing whether CCR2^+^ monocytes infiltrate the CNS of ALS mice. We used transgenic mice carrying a high copy number of a transgene encoding a G93A mutant of human superoxide dismutase 1 (SOD1^G93A^ Tg mice; #004435, Jackson Laboratory) as an ALS model. We obtained *Ccr2*^*rfp/+*^-*Cx3cr1*^*gfp/+*^-SOD1^G93A^ Tg mice by crossing SOD^G93A^Tg mice with *Ccr2*^*rfp/rfp*^ mice (#017586, Jackson Laboratory) and *Cx3cr1*^*gfp/gfp*^ mice (#005582, Jackson Laboratory), which enables us to easily differentiate CCR2^+^ infiltrating monocytes from CX3CR1^+^ tissue-resident macrophages (i.e., microglia in the CNS) [2, 6]. These mice were backcrossed for more than 10 generations after purchase. Histological analysis was carried out using 20-μm-thick frozen sections of lumbar spinal cords in *Ccr2*^*rfp/+*^-*Cx3cr1*^*gfp/+*^-SOD1^G93A^ Tg mice. Sections were permeabilized with 1% Triton X-100 after blocking with 10% normal goat serum for 30 min, and then stained with specific primary antibodies for markers of neurons (NeuN; #MAB377, Chemicon, Temecula, CA, USA), monocytes/microglia (Iba1; #019–19,741, Wako, Osaka, Japan), and astrocytes (GFAP; #M0761, Dako, Glostrup, Denmark), followed by Alexa Fluor 488–conjugated secondary antibodies (Molecular Probes, Eugene, OR, USA). Images were analyzed on a deconvolution fluorescence microscope (BZ-9000, Keyence, Osaka, Japan), and quantitative analyses of immunopositive cells in the lumbar spinal cord (6 sections/mouse, *n* = 5) were carried out using ImageJ as described [7]. Statistical significance was determined by one-way analysis of variance (ANOVA) followed by post-hoc Tukey’s test using Prism version 7.0 (GraphPad Software, La Jolla, CA, USA).

**Fig. 1:**
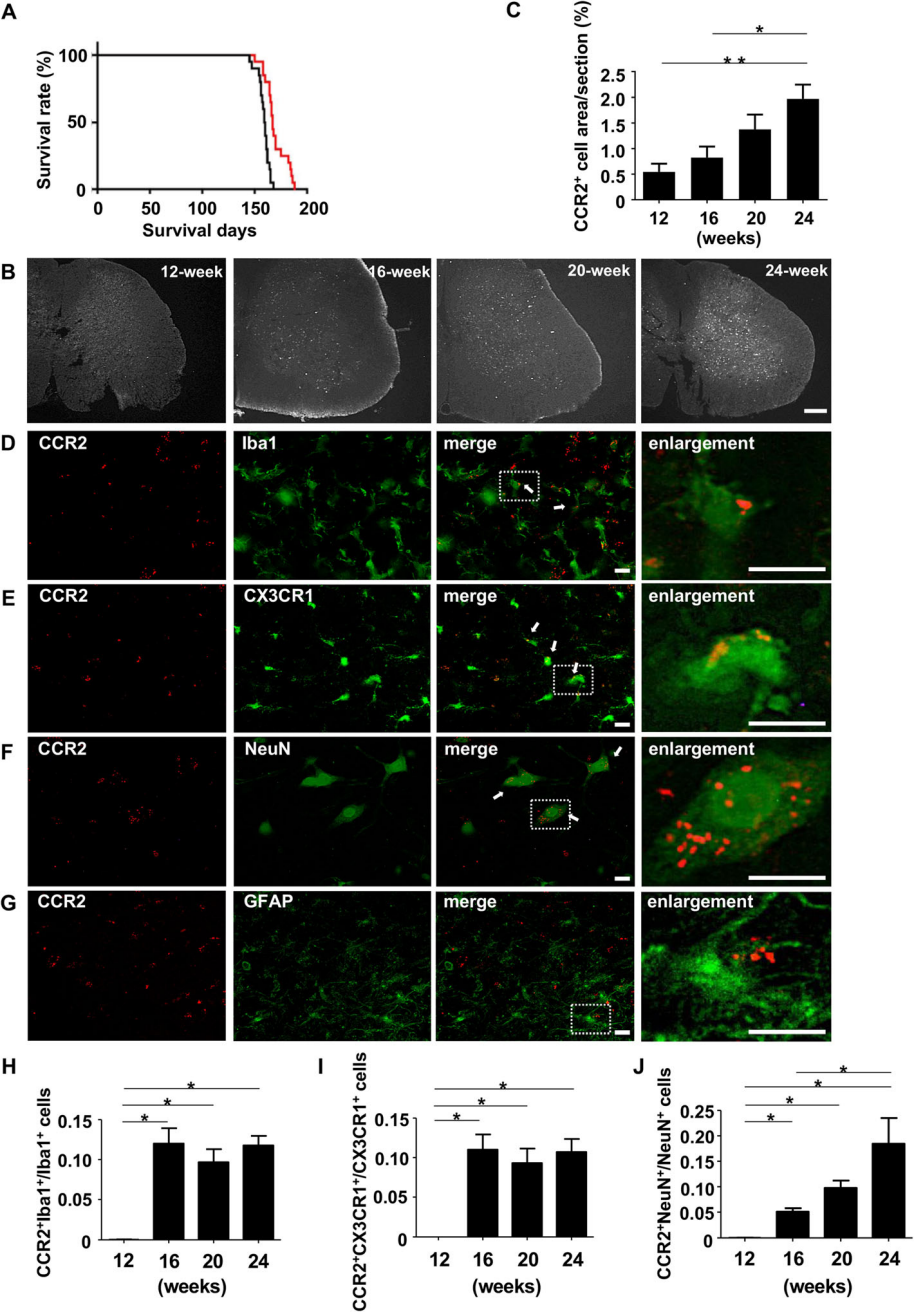
CCR2^+^ cells in the lumbar spinal cord of ALS mice. **a** Survival curve of mice. Red line, *Ccr2*^*rfp/+*^-*Cx3cr1*^*gfp/+*^-SOD1^G93A^ Tg mice; black line, SOD1^G93A^Tg mice (*n* = 20 in each group). **b** Representative low-magnification microscopic images of L5 lumbar spinal cord of *Ccr2*^*rfp/+*^-*Cx3cr1*^*gfp/+*^-SOD1^G93A^ Tg mice. The abundance of CCR2^+^ cells increased over the course of disease progression (12-week, early stage; 16-week, middle stage; 20-week, late stage; 24-week, end stage). Scale bar, 100 μm. **c** Percentage of CCR2^+^ cells area in L5 lumbar spinal cord (*n* = 5). *, *p* < 0.05. **, *p* < 0.01. **d**–**g** Immunofluorescence micrographs of L5 lumbar spinal cords in 24-week, end-stage ALS mice revealed that CCR2 (red) was localized in (**d**) infiltrating monocytes and microglia (Iba1, green), (**e**) microglia (CX3CR1, green), and (**f**) neurons (NeuN, green), but not in (**g**) astrocytes (GFAP, green). The right panels show enlargements of the dotted areas in the merged images in D–G. Scale bar, 10 μm. **h**–**j** Percentage of CCR2^+^ cells for (H) Iba1^+^ infiltrating monocytes and microglia, (**i**) CX3CR1^+^ microglia, and (**j**) NeuN^+^ neurons (12-week, early stage; 16-week, middle stage; 20-week, late stage; 24-week, end stage). *, *p* < 0.05

*Ccr2*^*rfp/+*^-*Cx3cr1*^*gfp/+*^-SOD1^G93A^ Tg mice exhibited similar disease progression to SOD1^G93A^ Tg mice (median survival days = 167 vs. 160, respectively; Fig. 1), and the abundance of CX3CR1^+^ microglia in lumbar spinal cord gradually increased as the disease progressed (Supplemental Figure 1), consistent with the previous reports [7, 8]. Chronological assessments also revealed that the abundance of CCR2^+^ cells gradually increased in the gray matter of the lumbar spinal cord as disease progressed (Fig. 1b and c), whereas CNS infiltration by CCR2^+^ cells was not detected in *Ccr2*^*rfp/+*^-*Cx3cr1*^*gfp/+*^ mice. Next, we evaluated the cellular localization of CCR2 by immunofluorescence staining for NeuN (neuron marker), Iba1 (monocytes and microglia marker), and GFAP (astrocyte marker). Most of CCR2^+^ cells were positive for Iba1 (Fig. 1d and h), but not for CX3CR1, until the middle stage of the disease, suggesting that most CCR2^+^ cells in the CNS were infiltrating monocytes at early disease stages. These findings corresponded with a previous study reporting that CCR2^+^ monocytes were recruited into the spinal cord of SOD1^G93A^Tg mice [3]. Surprisingly, from the middle stage of the disease, CCR2 was partially distributed in CX3CR1^+^ microglia (Fig. 1e and i) and neurons (Fig. 1f and j), but not in astrocytes (Fig. 1g), in striking contrast to the previous findings by Kawaguchi-Niida et al. [5]. The proportion of each type of CNS-resident cell that was CCR2^+^ increased as disease progressed, whereas the percentage of CX3CR1^+^ or Iba1^+^ cells that was CCR2^+^ reached a plateau at the middle stage of the disease (Fig. 1i). No resident CNS cells expressed CCR2 in *Ccr2*^*rfp/+*^-*Cx3cr1*^*gfp/+*^ non-Tg mice (Supplemental Figure 2). These novel observations demonstrated that CCR2 is expressed in resident CNS cells such as microglia and neurons, as well as CNS-infiltrating monocytes, in the advanced stage of ALS.

The cellular distribution and physiological role of CCR2 in the CNS have yet to be elucidated. Previous studies reported that CCR2 is present only in monocytes/macrophages and basophils, whereas *Ccr2* mRNA is expressed in most leukocytes, including monocytes/macrophages, T cells, B cells, natural killer cells, basophils, and dendritic cells [2]. However, CCR2 is constitutively expressed in neurons in murine brain, spinal cord, and dorsal root ganglia, and upregulation of CCL2 (the ligand of CCR2)–CCR2 axis in the disease state directly causes neuronal dysfunction through Akt signaling pathway [9–11]. Other studies reported that CCR2 is expressed in both infiltrating monocytes and microglia in a rodent model of traumatic brain injury [12]. Furthermore, another study reported CCR2^+^ monocyte infiltration in the perivascular areas of the primary motor cortex in ALS patients with TDP-43 pathology [13]. These data are discordant with the findings of Kawaguchi-Niida et al., who reported CCR2 expression exclusively in astrocytes [5]. Also in our ALS mice, CCR2-RFP was not detected in astrocytes, but was instead found in CNS-infiltrating monocytes, CX3CR1^+^ microglia, and neurons.

There are two possible origins of CCR2^+^CX3CR1^+^ cells in the spinal cord of ALS mice. One possibility is that CX3CR1^+^ microglia express CCR2. Initially, CCL2 is released from activated microglia and recruits CCR2^+^ monocytes into the spinal cord. Infiltrated CCR2^+^ monocytes also release CCL2, which accelerates inflammatory cell accumulation and leads to environmental deterioration including neuroinflammation and neuronal dysfunction, further provoking neuronal CCR2 expression. Subsequently, this microenvironmental change might convert CX3CR1^+^ microglia to CCR2^+^CX3CR1^+^ microglia acquiring deleterious phenotype as toxic conversion (i.e., CCR2 as a marker of neuroinflammation). Finally, the combination of CCR2^+^ cells (infiltrating monocytes, microglia, and neurons) might form a vicious cycle of neuroinflammation through CCL2–CCR2 signaling, thereby accelerating ALS pathology, in accordance with previous findings [1, 3, 12]. In fact, recent microglial transcriptional analyses demonstrated that the TREM2–APOE pathway induces dysregulation of *Cx3cr1* and homeostatic signature genes such as *P2ry12*, *Siglech*, and *Tmem119* in a mouse model of neurodegenerative disease, suggesting that microglia are activated in a detrimental manner in neurodegenerative diseases [14]. Our observations also indicated that the phenotypic conversion of homeostatic CX3CR1^+^ microglia to disease-associated CCR2^+^CX3CR1^+^ microglia might contribute to disease progression of ALS.

The other possibility is that CNS-infiltrating CCR2^+^ monocytes express CX3CR1. A previous study reported that chronic brain injury causes CX3CR1 upregulation in infiltrating CCR2^+^ monocytes, and that CCR2^+^CX3CR1^+^ monocytes control their own inflammation via neuronal CX3CL1 signaling [15]. Therefore, CCR2^+^CX3CR1^+^ monocytes may act as a self-limiting system of neuroinflammation in advanced ALS. Further studies are needed to elucidate the precise roles and mechanisms of CCR2^+^ cells in ALS pathology.

In conclusion, using a *Ccr2*^*rfp/+*^-*Cx3cr1*^*gfp/+*^ mouse, we revealed that CCR2 expression expands from CNS-infiltrating monocytes to resident CNS cells such as microglia and neurons, but not astrocytes, over the course of ALS disease progression. This reporter mouse represents a useful and powerful tool that could provide new insights into ALS pathomechanisms.

## Supplementary information

**Additional file 1 **Activation of CX3CR1^+^ microglia in the lumbar spinal cord of ALS mice. (A) Representative low-magnification microscopic images of L5 lumbar spinal cord of *Ccr2*^*rfp/+*^-*Cx3cr1*^*gfp/+*^-SOD1^G93A^ Tg mice. The abundance of CX3CR1^+^ microglia increased as disease progressed (12-week, early stage; 16-week, middle stage; 20-week, late stage; 24-week, end stage). Scale bar, 100 μm. (B) Percentage of CX3CR1^+^ microglia area in L5 lumbar spinal cord (*n* = 5). *, *p* < 0.05.

**Additional file 2 **Absence of CCR2 in the lumbar spinal cord of *Ccr2*^*rfp/+*^-*Cx3cr1*^*gfp/+*^ non-Tg mice. Immunofluorescence micrographs of L5 lumbar spinal cords in 24-week *Ccr2*^*rfp/+*^-*Cx3cr1*^*gfp/+*^ non-Tg mice demonstrated that CCR2 (red) was not localized in (A) infiltrating monocytes and microglia (Iba1, green), (B) microglia (CX3CR1, green), (C) neurons (NeuN, green), or (D) astrocytes (GFAP, green). Scale bar, 10 μm.

## Data Availability

The datasets used and/or analyzed in this study are available from the corresponding authors on reasonable request.

## Abbreviations

ALS: Amyotrophic lateral sclerosis
CNS: Central nervous system
SOD1: Superoxide dismutase 1
