# Supplementary figures and images for: CCR2 is localized in microglia and neurons, as well as infiltrating monocytes, in the lumbar spinal cord of ALS mice
Source: Mol Brain. 2020 Apr 29;13:64. doi: 10.1186/s13041-020-00607-3 (PMC7191738; doi:10.1186/s13041-020-00607-3)

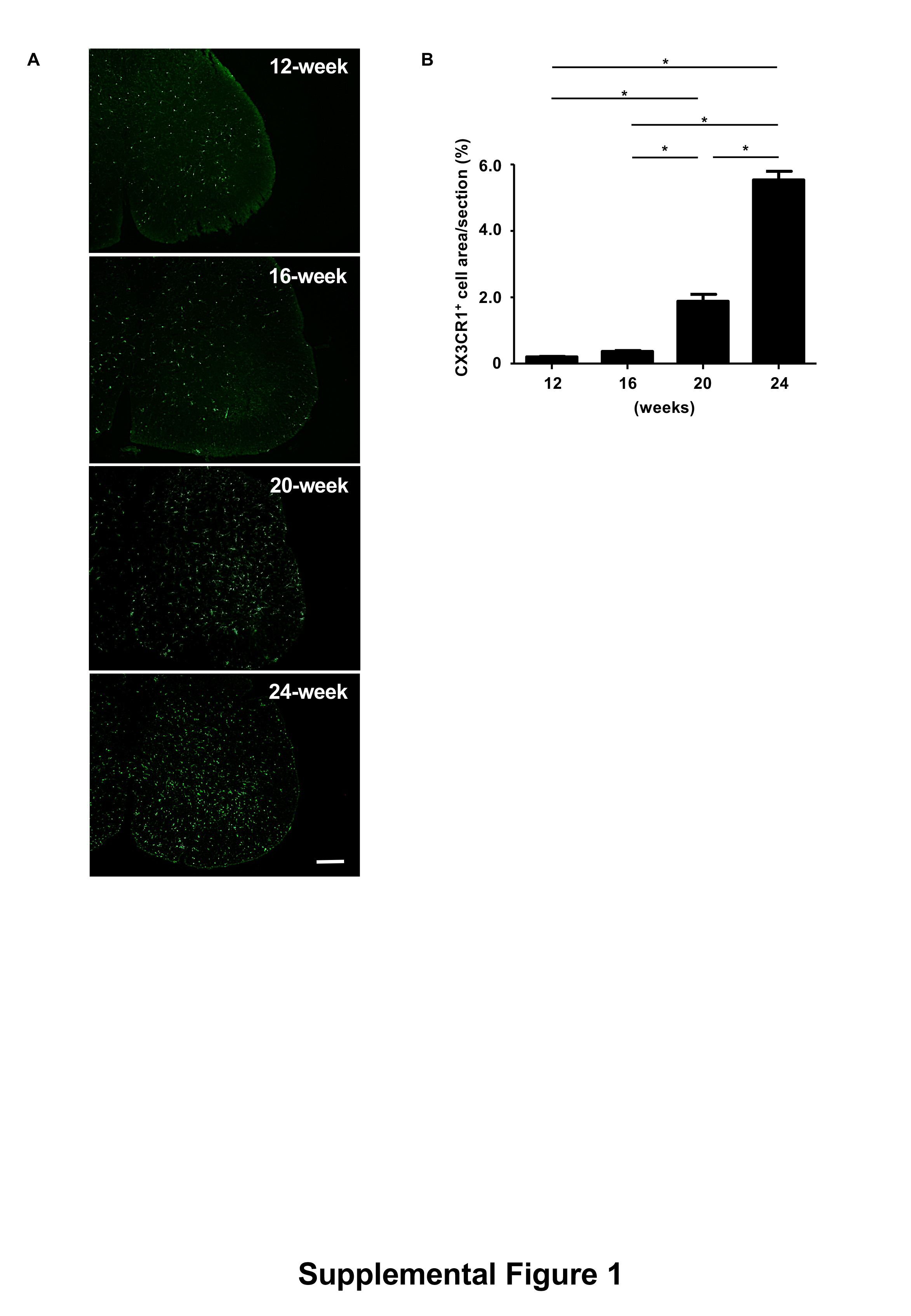

Supplement: Supplementary file 1 — Additional file 1 Activation of CX3CR1+ microglia in the lumbar spinal cord of ALS mice. (A) Representative low-magnification microscopic images of L5 lumbar spinal cord of Ccr2rfp/+-Cx3cr1gfp/+-SOD1G93A Tg mice. The abundance of CX3CR1+ microglia increased as disease progressed (12-week, early stage; 16-week, middle stage; 20-week, late stage; 24-week, end stage). Scale bar, 100 μm. (B) Percentage of CX3CR1+ microglia area in L5 lumbar spinal cord (n = 5). *, p < 0.05. [file 13041_2020_607_MOESM1_ESM.tif]

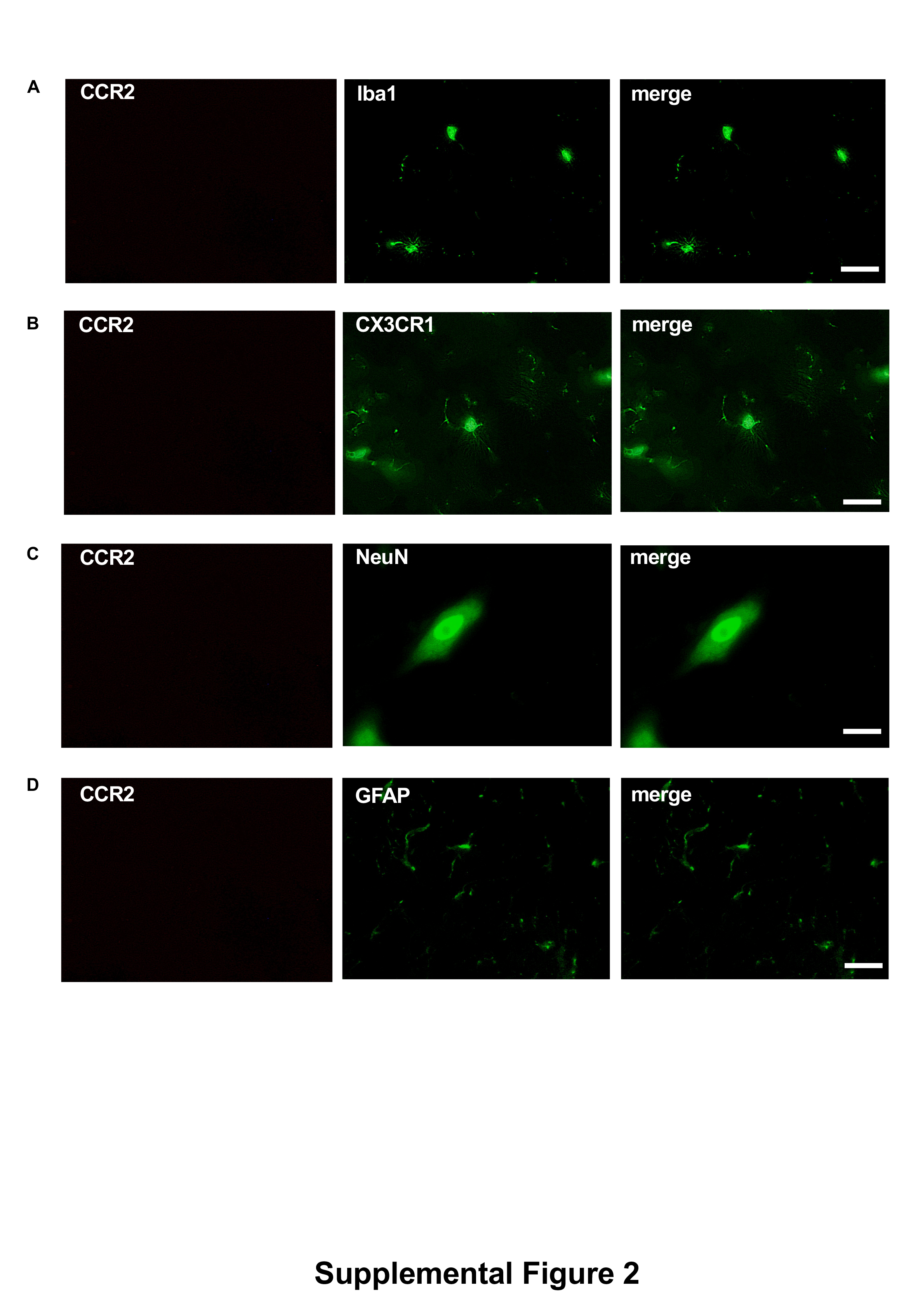

Supplement: Supplementary file 2 — Additional file 2 Absence of CCR2 in the lumbar spinal cord of Ccr2rfp/+-Cx3cr1gfp/+ non-Tg mice. Immunofluorescence micrographs of L5 lumbar spinal cords in 24-week Ccr2rfp/+-Cx3cr1gfp/+ non-Tg mice demonstrated that CCR2 (red) was not localized in (A) infiltrating monocytes and microglia (Iba1, green), (B) microglia (CX3CR1, green), (C) neurons (NeuN, green), or (D) astrocytes (GFAP, green). Scale bar, 10 μm. [file 13041_2020_607_MOESM2_ESM.tif]
